# Bone, a Secondary Growth Site of Breast and Prostate Carcinomas: Role of Osteocytes

**DOI:** 10.3390/cancers12071812

**Published:** 2020-07-06

**Authors:** Paola Maroni, Paola Bendinelli

**Affiliations:** 1 IRCCS Istituto Ortopedico Galeazzi, Laboratory of Experimental Biochemistry & Molecular Biology, Via R. Galeazzi 4, 20161 Milano, Italy; 2 Dipartimento di Scienze Biomediche per la Salute; Università degli Studi di Milano, Via L. Mangiagalli 31, 20133 Milano, Italy; paola.bendinelli@unimi.it

**Keywords:** osteocytes, breast cancer, prostate cancer, bone metastasis, sclerostin, Dickkopf-1(DKK-1), fibroblast growth factor 23 (FGF23)

## Abstract

Bone is the primarily preferred site for breast and prostate cancer to metastasize. Bone metastases are responsible for most deaths related to breast and prostate cancer. The bone’s particular microenvironment makes it conducive for the growth of cancer cells. Studies on bone metastasis have focused on the interaction between cancer cells and the bone microenvironment. Osteocytes, the most common cell type of bone tissue, have received little attention in bone metastasis, although they are master signal sensors, integrators, and skeleton transducers. They play an important role in regulating bone mass by acting on both osteoblasts and osteoclasts, through the release of proteins such as sclerostin, Dickkopf-1 (DKK-1), and fibroblast growth factor 23 (FGF23). Osteocytes have been extensively re-evaluated, in light of their multiple functions: with different experimental approaches, it has been shown that, indeed, osteocytes are actively involved in the colonization of bone tissue by cancer cells. The present review focuses on recent research on the role that osteocytes play in bone metastasis of breast and prostate cancers. Moreover, the studies here summarized open up perspectives for new therapeutic approaches focused on modulating the activity of osteocytes to improve the condition of the bone metastatic patients. A better understanding of the complex interactions between cancer cells and bone-resident cells is indispensable for identifying potential therapeutic targets to stop tumor progression and prevent bone metastases.

## 1. Introduction

Metastases are by far the most common malignant neoplasms of the skeleton. Bone colonization by breast and prostate cancer cells is a frequent complication of these cancers, two of the main gender-related tumor types. Overall, the world population has a 20.2% risk of developing cancer (22.4% in men and 18.2% in women aged 0 to 74 years): the risk of breast cancer in women is 5.03% and the risk of prostate cancer in men is 3.73% [1]. Treatment for breast and prostate cancer has improved life expectancy. The 5-year overall survival is 70–100% for prostate cancer and 80–85% for breast cancer, which drop to about 30% in metastatic cancers [1]. More than 70% of patients with late-stage breast or prostate cancer will develop skeletal metastasis, which is considered a terminal illness. Moreover, such patients frequently manifest skeletal-related events (SREs), including aberrant bone remodeling, abnormal fractures, bone pain, spinal cord compression, and hypercalcemia, which markedly reduce quality of life.

Knowledge of the molecular mechanisms by which breast and prostate cancer cells colonize bone tissue is essential for finding new therapeutic targets to halt tumor progression and prevent metastases to bone. With this in mind, studies investigating the crosstalk between metastatic cells and the cells that make up the bone microenvironment have underlined the importance of communication between cancer cells and osteoblasts and osteoclasts [2,3].

Even though osteocytes are the major constituent among skeletal resident cells, they were long considered marginal in bone metastasis. However, they are master signal sensors, integrators, and transducers of the skeleton, and they play a fundamental role in regulating bone mass by acting on both osteoblasts and osteoclasts. Their multiple functions have been re-evaluated and attracted growing attention. A role for osteocytes is beginning to emerge in osteolytic lesions derived from multiple myeloma, where the crosstalk between cancer cells and osteocytes decrease osteocyte viability [4]. This review summarizes the advances in current knowledge of osteocyte biology and of colonization of bone metastases derived from breast and prostate carcinomas.

## 2. Bone Metastasis from Breast and Prostate Carcinomas: Osteolytic and Osteoblastic Lesions

Cancer cells with malignant potential develop various strategies to reach secondary growth sites: they escape immunosurveillance [5,6], degrade the basement membrane and the extra cellular matrix (ECM), invade the surrounding microenvironment (local invasion), and enter the bloodstream and/or the lymphatic system, ultimately reaching sites where they will give rise to metastatic growth [3,7,8]. Bone metastasis differs in aspect depending on the interactions between the cancer cells and the complex tissue microenvironment. The crosstalk between metastasizing cancer cells and bone tissue components can give rise to osteolytic metastases, with bone destruction, and osteoblastic metastases, with the formation of bone or mixed lesions. Some types of neoplasms are associated with one or another pattern of bone metastases.

Breast cancer spreads in the bone and results mainly in osteolytic metastases (more rarely mixed metastasis), while osteoblastic bone metastasis is the hallmark feature of prostate cancer [9,10]. Bone lesions from prostate cancer can be blastic or lytic or mixed [11].

Osteolytic metastasis is characterized by a “vicious cycle” in which osteoclasts, activated directly or indirectly by tumor cells [12], increase in function and abnormal bone resorption, leading to the release from the bone matrix of growth factors, e.g., transforming growth factor β (TGF-β). These factors are important, because they stimulate tumor growth and osteoclast activation [13]. Parathyroid hormone-related protein (PTHrP), produced by cancer cells, enhances receptor activator of nuclear factor kappa B ligand (RANKL) expression and inhibits osteoprotegerin (OPG) secretion from osteoblasts and stromal cells, thus activating osteoclastogenesis via RANK located on osteoclast precursors [14].

A “vicious cycle” takes place also in osteoblastic lesions: cancer cells secrete factors that enhance angiogenesis and differentiation of mesenchymal stem cells into osteoblasts. In turn, osteoblasts induce osteoclast differentiation and activation; growth factors, released by bone resorption, stimulate the growth of tumor cells [15].

Factors derived from prostate cancer and the bone microenvironment target prostate cancer cells to bone and control cancer cell growth in the new site. Prostate cancer cells are attracted to bone regions rich in osteoblasts, where direct contact between metastatic prostate cancer cells and osteoblasts stimulates the proliferation of both cell types [16,17]. After the prostate cancer cells have settled in the bone, the lesions are initially osteolytic. During this phase, the prostate cancer cells interact with components of bone, cells, and matrix to produce osteoclast-activating factors. The resulting osteolysis makes available factors (which lie in the bone matrix, e.g., TGF-β, insulin-like growth factor-1 [IGF-1], matrix metalloproteinases [MMPs], fibroblast growth factor [FGF], bone morphogenetic proteins [BMPs], and platelet-derived growth factor [PDGF]) to stimulate prostate cancer cell proliferation. The osteolytic phase is also influenced by a plethora of factors derived from cancer cells and bone.

Moreover, prostate cancer cells can also produce osteoblastic lesions. During this phase, prostate cancer cells induce a dysfunctional osteoblastic phenotype, through the production of factors including BMPs, TGF-β, PDGF, adrenomedullin, IGF-1, FGF, and vascular endothelial growth factor (VEGF). The osteoblastic phase leads to the formation of hypermineralized bone with multiple layers of poorly organized type I collagen fibrils of reduced mechanical strength. Secretory kinases and phosphatases are also involved in the formation of aberrant mineralization that makes phosphate and calcium available and regulates the function of proteins relevant for mineralization [18].

Even though osteoblast proliferation increases in metastatic prostate cancer, the new bone is not compact and lamellar, but rather spongeous in structure due to misalignment of osteoblasts along the collagen matrix. This poorly organized bone matrix reduces bone strength and function [19,20]. In addition, osteoblast stimulation and the secretion of endothelin-1 (ET-1) by prostate cancer cells have been demonstrated to suppress Dickkopf-1 (DKK-1), which stimulates Wingless-related integration site (Wnt) signaling and osteoblast bone deposition [21]. This mechanism is characteristic of tumor cells in the bone microenvironment.

Bone is a dynamic tissue; it is essential for structural support, movement, and storage of minerals and energy. Diverse cell populations reside within the bone: bone-remodeling cells, osteoblasts, and osteoclasts, cells that maintain structural integrity and bone health; osteocytes that, in response to mechanical cues and systemic hormones, regulate bone remodeling [22].

## 3. Osteocytes: Their Physiology and Possible Contributions to Bone Metastasis

### 3.1. Osteocytes in Bone Physiology

Embedded in the bone matrix, osteocytes make up about 90–95% of adult bone cells. Osteocytes differentiate from osteoblasts (which derive from mesenchymal stem cells) through a multiphase process, in which each step is governed by key molecular regulators [23]. The ontogenesis of osteocytes starts with the transformation of polygonal cells, osteoblasts, into branched cells with dendritic processes that extend toward the mineralizing front. During the multi-step differentiation, the osteoid osteoblasts/osteoid osteocytes are embedded in the cortical bone control and regulate the bio-mineralization and the formation of connective dendritic processes in a prelude to the formation of mineralizing osteocytes. Finally, when maturation is complete, mature osteocytes secrete sclerostin, a negative regulator of bone formation [24,25].

Via their numerous dendritic processes, the osteocytes come into contact with each other and interact with other cell types to facilitate the distribution of autocrine/paracrine secreted factors and the coordination of bone responses to mechanical and biological stimuli [26]. The connections between the lacunar–canalicular and the blood vessel networks allow the exchange of small molecules [27]. The extracellular space within the canaliculi is accessible to peripheral blood vessels. Osteocytes are immersed in a canalicular fluid that supplies them with nutrients, oxygen, and information from systemic circulation. The canalicular fluid transports hormones, exchanges circulating factors, delivers mechanical signals, and allows access to potential therapeutic drugs [28].

The system is composed of osteocyte, lacunar, and canalicular networks; it acts as a mechanical strain amplifier to make the cells more sensitive to mechanical loading. Because osteocytes are extensively interconnected in the bone matrix, they can translate mechanical load into biochemical signals and influence bone remodeling by orchestrating formation and resorption signals. By means of cellular sensors (e.g., integrins, cilium, calcium channels, and G-protein-coupled receptors) osteocytes can sense mechanical forces and respond through the release of factors that stimulate effector cells [29]. Besides acting as a mechanosensors, osteocytes control osteoblasts through the release of sclerostin and control osteoclasts via the RANK/RANKL/OPG pathway.

Sclerostin is a product of the *SOST* gene; it is mainly produced by osteocytes and to a lesser degree by other tissues such as cartilage, kidney, heart, and liver [30,31,32]. Sclerostin regulates normal bone remodeling: after binding at the low-density lipoprotein receptor-related protein 5/6 (Lrp5/6), it reduces bone formation by inhibiting the Wnt/βcatenin pathway in osteoblasts or induces osteoblastic cell apoptosis by activating caspases. In normal bone physiology, sclerostin acts on the surface of bone trabeculae, and its expression depends on mechanical loading, inflammatory molecules (i.e., prostaglandin E2, PGE2), and hormones (i.e., PTH and estrogen) [33]. Sclerostin levels are decreased in the skeletal areas where tension is concentrated due to the release of prostaglandins by osteocytes. In vitro studies have shown that PGE2 rapidly inhibits sclerostin via the EP2 receptor [34].

The osteocyte lacuno-canalicular network is also intimately associated with the blood vessel network in the bone matrix. Angiogenesis, which is necessary for bone remodeling, is affected also by osteocytes which, via the signaling pathways of VEGF and mitogen-activated protein kinases (MAPKs), confer angiogenic properties to endothelial cells [35]. The production by osteocytes of angiogenic factors, such as VEGF [33], RANKL [36], and BMP7 [37,38], is relevant for initiating bone remodeling. In vitro studies have demonstrated that sclerostin has angiogenetic properties: it guides the formation of new vessels that activate the recruitment of osteoclasts and their precursors which, like monocytes, leave the bloodstream and arrive at bone reabsorption sites [39]. A role for osteocytes in tumor-associated angiogenesis has not yet been shown, however.

### 3.2. Osteocytes in Bone Metastasis from Breast and Prostate Carcinomas: Active Players

The role of osteocytes in bone metastases is underestimated, which is why their role in tumor cell invasion and metastases remains poorly defined. One of the first observations of active participation of osteocytes in metastasis reported that the production by osteocytes of CXCL12 stimulates activation of the CXCL12-CXCR4 signaling axis in cancer cells and promotes their homing to bone [40]. Recent studies investigating whether osteocytes influence breast and prostate cancer cell behavior showed that human prostate cancer cell lines (PC-3 and DU145) and human breast cancer cell lines (MDA-MB-231 and MCF-7) treated with conditioned medium obtained from murine osteocyte cell line MLO-Y4 cells were stimulated to proliferate [41]. Conditioned medium from murine osteocyte cell line MLO-A5 cells was able to increase the proliferation of murine breast cancer cell line 4 T1.2 cells [42,43]. Hence, osteocytes appear to have an impact on tumor growth.

Chen and colleagues demonstrated the potential role of osteocytes in bone metastasis by studying tumor–osteocyte interactions using two cell lines, MDA-MB-231 breast carcinoma cells, and osteocytic cells MLO-A5/MLO-Y4. In the three-dimensional spheroid assay, tumor spheroids significantly shrank in the presence of co-cultured osteocyte spheroids: osteocytes attract and compact tumor cells [42]. In vitro analysis revealed that tumor-osteocyte interactions down-regulated the expression of Snail, a factor involved in epithelial-to-mesenchymal transition (EMT), raising the hypothesis that the tumor-osteocyte interaction induces the reversal of EMT of breast cancer cells in the bone microenvironment [43]. The mesenchymal-to-epithelial transition (MET) takes place in secondary growth sites and is important for colonization [44]. In the bone microenvironment, osteocyte-secreted bone matrix proteins attract and compact migratory breast cancer cells and suppress tumor migration by Snail down-regulation, thus promoting tumor growth and metastatic colonization [43].

Mei and coworkers developed a novel microfluidic platform to study breast cancer extravasation in bone: the device reconstructs cellularized 3D microfluidic tissue, in which three different cell types (osteocytes, breast cancer, and human umbilical vein endothelial cells [HUVEC]), interact under mechanical stimulation. Their findings suggest that mechanically stimulated osteocytes inhibit transendothelial breast cancer extravasation: both extravasation distance and percentage of extravasated side channels were far more reduced in the mechanically stimulated osteocytes than in the static osteocytes [45].

Accordingly, in vitro studies have shown that mechanical stimulation of osteocytes reduces the transendothelial migration of breast cancer cells and that the osteocyte–endothelial cell interaction inhibits the expression of matrix metalloproteinase 9 (MMP-9), an enzyme involved in the invasion of metastatic cancer cells through the extracellular matrix [46]. Further, mechanical stimulation of osteocytes affects breast cancer cells, not only through direct signaling, but also through osteoclasts and endothelial cells [47].

The effect of mechanical stimulation of osteocytes on breast cancer cell behavior has been further investigated [48]. In the absence of mechanical stimulation, osteocytes suppress tumor migration by down-regulating the expression of Src and Snail. In an in vivo mouse model of tibial osteolysis, mechanical stimulation had intensity-dependent effects on the tumor cell: a low mechanical load increased osteopontin (OPN) and inhibited tumor migration, while a medium level reduced OPN and stimulated tumor migration. The researchers concluded that while mechanical load on the skeleton could be beneficial for the prevention of bone metastases from breast cancer, the load intensity should be carefully monitored [48]. In another study analyzing how breast cancer cells modulate dendrite formation and function of osteocytes, skeletal mechanical loading was found to stimulate breast cancer cells to alter osteocyte mechanosensing and bone remodeling by increasing dendrite formation and downstream resorption. It was speculated that osteocytes could serve as a cellular link between mechanical loading and breast cancer-induced bone disease [49].

Wang and colleagues proposed a novel mechanism through which prostate cancer cells educate osteocytes to promote tumor progression and bone metastasis. Through in vitro and in vivo experiments, they demonstrated that prostate cancer cells induce osteocytes to produce growth-derived factor 15 (GDF15) which, in turn, enhanced the expression of the early growth response 1 (EGR1) transcription factor in prostate cancer cells and regulated the growth and the invasive properties of cancer cells in a positive feedback loop [50].

Moreover, it has been demonstrated that the growth of prostate cancer cells in bone increases tumor-generated pressure and modifies the bone microenvironment, so as to promote the spread of bone metastases. Osteocytes, as mechanotransducers in the bone, sense these changes in physical force and modify their function. In this regard, osteocytes exposed to hydrostatic pressure were observed to reproduce conditions that occur in vivo when tumor growth creates intraosseous pressure. The experiments revealed that treatment enabled osteocytes to promote viability, motility, and invasion of prostate and breast cancer cells through the release of Regulated upon Activation, Normal T cell Expressed and Secreted (RANTES) and MMPs [51]. Osteocytes can participate with osteoclasts and osteoblast in the “vicious cycle” by secreting pro-osteoclastogenic cytokines and stimulating bone resorption, thus altering bone homeostasis and fueling tumor growth.

Osteocytes are long-living cells with a life span of up to 25 years; they die by apoptosis, a mechanism spatially, temporally, and functionally linked to bone remodeling [52]. Apoptotic osteocytes are targeted for bone resorption, which increases bone turnover [53]. Apoptosis of osteocytes was shown to precede osteoclastic activity, thus suggesting that it generates a signal in targeted osteoclastic invasion. Also, apoptotic osteocytes are important contributors to abnormal bone resorption, which drives the development of cancer-induced osteolytic lesions [24,54].

Chemotherapy has direct cytotoxic effects on bone cells: it increases osteoclast activity and inhibits osteoblast activity, and induces osteocyte apoptosis [55]. In Vivo and in vitro experimental models have shown that methotrexate, a chemotherapy drug, induces osteocyte apoptosis and the release of factors that significantly stimulate osteoclastogenesis [56].

Apoptotic osteocytes are increased in patients with multiple myeloma; they produce high levels of IL-11, which can stimulate osteoclast formation, and they augment their expression of IL-11 after a bone lesion develops [4]. What role osteocyte apoptosis plays in metastasis from breast and prostate cancer is not known, though IL-11 is critically involved in osteolysis in metastatic tissue [57].

The physical interaction between osteocytes and myeloma cancer cells induces activation of Notch signaling: osteocytes accelerate the proliferation of myeloma cells by increasing cyclin D1 mRNA expression and Notch stimulates rapid programmed cell death (apoptosis) in osteocytes [58]. Hence, targeting Notch signaling in the metastatic niche could be an important approach to reduce tumor growth, maintain osteocyte viability, and inhibit bone destruction. To avoid off-target effects, it would be of interest to target specific components of the Notch pathway. Nevertheless, further studies are needed to define the specific effects of Notch signaling on osteocytes and other cancers and to determine whether Notch pathway targeting is effective in the treatment of bone cancer [59].

Therefore, with various experimental approaches, it has been shown that osteocytes can condition the behavior as well as the phenotype of cancer cells and affect metastatic colonization by acting in different stages of the process.

Table 1 summarizes the crosstalk between osteocytes and breast/prostate cancer cells.

### 3.3. Sclerostin, Dickkopf-1 (DKK-1), and Fibroblast Growth Factor 23 (FGF23) in Breast and Prostate Cancer: New Therapeutic Opportunities?

Sclerostin, DKK-1, and FGF23 are osteocyte-specific markers; they were studied to evaluate their contribution to osteocyte/cancer cell crosstalk [24,33,60]. DKK-1 and sclerostin are negative regulators of the Wnt/β-catenin pathway; sclerostin is expressed almost exclusively in osteocytes at high levels, while DKK-1 is present throughout the body. FGF23 is normally expressed by osteocytes and plays a critical role in calcium and phosphate homeostasis [61].

Sclerostin acts by targeting osteoblasts during their differentiation to mature osteocytes to inhibit bone mineralization and to up-regulate the expression by osteocytes of catabolic factors that results in osteocytic osteolysis [62]. Moreover, sclerostin is able to up-regulate the expression of RANKL by osteocyte-like cells and to promote osteoclastogenesis [63], supporting the view that osteocytes are targets for the action of sclerostin [64]. The implication of sclerostin in the biology of bone loss in bone disorders and in malignant diseases like breast or prostate cancer has attracted research interest in this direction.

Breast cancer cells undergo a phenotypic change in the bone by assuming osteomimetic properties [65], including the ability to release sclerostin. Once released, sclerostin inhibits osteoblast function; in prostate cancer, which predominantly produces osteoblastic bone metastases, sclerostin levels are low [66]. Accordingly, sclerostin could be a promising factor for the therapy of osteolytic bone metastasis from breast and myeloma-related bone disease [66].

Zhu and colleagues reported that sclerostin was over-expressed in breast cancer tumor tissues and cell lines and that the inhibition of sclerostin by specific antibody significantly reduced migration and invasion of breast cancer cell lines in a time-and dose-dependent manner. In a xenograft model of bone metastasis, established using MDA-MB-231 cells implanted into the femur of nude mice, the researchers reported that the inhibition of sclerostin significantly improved overall survival and prevented osteolytic lesions [67].

A study performed in mice with breast cancer bone metastases secreting sclerostin, pharmacological treatment with an anti-sclerostin antibody (Setrusumab) was shown to reduce bone metastatic burden, protect against breast cancer-induced bone destruction, and inhibit tumor cell dissemination to other distant sites. Moreover, inhibition of sclerostin also prevented muscle fiber atrophy and muscle weakness, which frequently accompany metastatic breast cancer. The results indicate the usefulness of sclerostin inhibition as a therapeutic option for breast cancer patients [68].

Other interesting studies on sclerostin were focused on the role of miR-218 in metastatic bone disease from breast cancer [69,70]. This short non-coding microRNA promotes metastasis-related molecular properties in MDA-MB-231 aggressive breast cancer cells, by targeting sclerostin and secreted frizzled-related protein 2 (SFRP2), which greatly enhances Wnt signaling. The use of an antimiR-218-5p demonstrated the inhibition of tumor growth in the bone marrow microenvironment and metastatic spread. MiR-218 might act on sclerostin produced by breast cancer cells to reduce osteoblast function and differentiation.

Anti-sclerostin drugs have been tested in patients with low bone mass, and the results of phase I and II trials [71,72,73] opened the way to the clinical investigation of these agents in cancer-induced bone disease.

Among the Wnt signaling inhibitors, DKK-1 has attracted attention, due to its effect on the invasive and metastatic potential of cancer cells [74]. Based on its ability to inhibit Wnt, DKK-1 was originally characterized as a tumor suppressor. However, subsequent studies also reported its role as a tumor promoter. The ability of DKK-1 to act as a tumor suppressor or promoter depends on numerous contextual factors (e.g., type of cancer, heterogeneity within the tumor, and tumor microenvironment), indicating that the analysis of this system is complex for in vitro studies [75]. DKK-1 has been shown to inhibit invasion and migration in breast cancer [76] and in PC3 cells [77].

Wnt inhibitors seem to be involved in the progression of prostate cancer, with the development of bone metastases, and in castration-resistant prostate cancer. DKK-1 may play a role in mediating the inhibition of osteoblasts in the growth of osteoblastic metastases. However, sclerostin and DKK-1 levels may vary during disease progression, adding complexity to our knowledge of the process [78].

DKK-1 expression is elevated in prostate cancer tissue compared to benign tissue and seems to be associated with worse survival [79]. By contrast, the down-regulation of DKK-1 correlates with a delay in the development of bone metastases in prostate cancer [80]. Furthermore, elevated DKK-1 expression is an early event in prostate cancer; its expression declines with tumor progression, and this effect is particularly evident in advanced bone metastases. It has been reported that in some disease states, in which osteoblast number or osteoblastic activity is increased, such as Paget’s disease of bone and prostate cancer, sclerostin levels were found to correlate with the rate of bone turnover [81]. These data were explained as a compensatory system, by which osteocytes release sclerostin to down-regulate an overwhelming level of osteoblast activity [33]. Another interpretation suggests that sclerostin may induce osteocytic RANKL production and up-regulate osteoclast activity and bone resorption [63].

In their study, Choudhary and colleagues used a three-dimensional engineered tissue model in a microfluidic perfusion device to replicate the lacunar–canalicular structure created by osteocytes and the functions of human bone tissue. They studied the changes in the osteocytes induced by the arrival of prostate cancer cells to the bone [82]. They found that osteocytes modify their phenotype and show decreased expression of sclerostin and increased levels of DKK-1 in the presence of conditionally reprogrammed primary human prostate cancer cells. The researchers speculated that this event affects lesion progression: the decrease in sclerostin may promote osteoblastic lesions, while DKK-1 may act as a “molecular switch” towards osteolytic lesions, as reported by Sottnik et al. [83].

Choudhary et al. reported for the first time that the presence of prostate cancer cells significantly stimulates the expression of FGF23 by osteocytes [82]. Interestingly, other researchers suggested that FGF23 may play a role in cancer [84,85,86]. For example, FGF23 may act as an autocrine, paracrine and/or endocrine growth factor in prostate cancer progression, but the relative importance of each factor depends on tumor expression level, tumor site, and underlying disease state [85].

Mansinho et al. assigned a negative prognostic meaning to FGF: low baseline serum FGF23 levels correlated with longer overall survival and onset of SREs in metastasis-bearing patients [84].

Together, all these observations open up perspectives for new therapeutic approaches focused on modulating the activity of osteocytes to improve the condition of the bone-metastatic patients. In particular, the use of anti-sclerostin drugs has already passed phases I and II in other pathologies, but also DKK-1 and FGF23 appear promising targets and deserve further studies.

**Figure 1:**
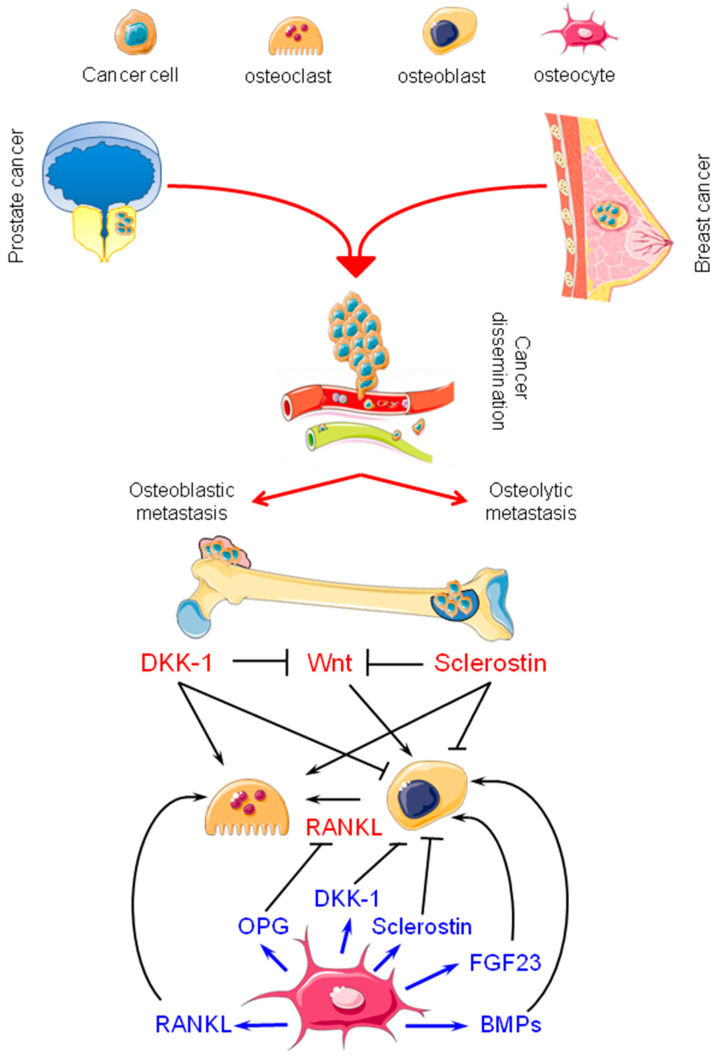
Overview of the effects of osteocyte products on osteoclasts and osteoblasts during bone metastasis. Also shown are the effects on osteoblasts and osteoclasts of sclerostin and DKK-1 produced by breast and prostate cancer cells at the metastatic site. DKK-1: Dickkopf-1; Wnt: Wingless-related integration site; RANKL: receptor activator of nuclear factor kappa B ligand; OPG: osteoprotegerin; FGF23: fibroblast growth factor 23; BPM: bone morphogenetic protein. (Figure created, with small modifications, using Servier Medical Art available at https://smart.servier.com).

Figure 1 summarizes the participation of osteocytes in metastatic colonization through the production of molecules capable of modulation both the activity of cancer cells and cells of bone microenvironment.

### 3.4. Cx43 Hemichannels: A New Way to Explore

Connexin (Cx) proteins form both gap junction channels and hemichannels: gap junction channels mediate intercellular communication between adjacent cells, whereas hemichannels permit the exchange of molecules between cells and extracellular environments. Cx43 is a protein recruited in the formation of gap junctions and it is the most abundantly expressed and studied subtype in bone.

In osteocytes, Cx43 is abundantly expressed and mediates communication among osteocytes and between osteocytes and other cells on the surface of bone trabeculae; Cx43 hemichannels are physiologically mechanosensitive and are activated by fluid flow and shear stress [87]. Cx43 hemichannels in osteocytes have low activity under normal conditions. In response to stimuli, such as bisphosphonates (therapeutical stimuli) and mechanical loading (physiological/pathological stimuli), they release molecules such as PGE2 and adenosine triphosphate (ATP). The activity of osteocytic Cx43 hemichannels has been demonstrated to play a key role in the inhibition of migration, invasion, and growth of breast cancer cells in the bone microenvironment. Mice lacking Cx43 in osteocytes showed a significant increase in breast cancer growth and resistance to zoledronic acid treatment [88]. These findings open exciting research avenues: the activity of Cx43 hemichannels could be exploited for new therapeutic strategies for the prevention of bone metastases and/or for their clinical treatment [88].

Moreover, a role for Cx43 in the regulation of apoptosis in osteocytes has been reported by Bivi et al. They showed that a Cx43 conditional knockout, in both mature osteoblasts and osteocytes, leads to an increase in osteocyte apoptosis, which is not observed when the knockout is performed in the early stages of osteoblast differentiation [89]. Dying osteocytes release apoptotic bodies that promote osteoclast recruitment and differentiation, thus triggering bone resorption [89]. These intriguing results highlight that further work is needed to determine whether and how hemichannels can be targeted to limit bone metastasis.

## 4. Conclusions

Osteocytes, long overlooked and regarded as cells trapped in the mineralized matrix with little importance in the development of bone metastases, have been recently re-evaluated. Indeed, osteocytes are not static, but rather highly dynamic cells: the dendritic interconnections between osteocytes and other cell types are not permanent. Since they can be “activated” or “deactivated” as needed, osteocytes may be able to redirect signaling between cells, based on the “switchboard” principle [90]. The crosstalk between osteocytes and osteoclasts and osteoblasts is also important in the development of bone metastasis; furthermore, osteocyte–cancer cell interaction is relevant for colonization. Such interaction is bidirectional: osteocytes sustain cancer cells in their consolidation in bone tissue, but their behavior depends, at least in part, on the signals received from cancer cells. These observations broaden the concept of the fertility of the metastatic soil typical of bone tissue and highlight how tumor cells are able to co-opt the microenvironment for their aims. Much remains to be done to understand the specific contribution of osteocytes to the relocation and homing of cancer cells to bone. Notions acquired through in vitro studies indicate the active participation of osteocytes in the metastatic process. In some cases, however, in vitro experiments are limited, because they cannot reproduce the bone microenvironment in its entirety, with the variety of cells that influence each other. Owing to this limitation, results only partially reflect the real situation. In any case, bone metastatic lesions, osteolytic or osteoblastic, are complexes and evolve by changing their characteristics. The contribution of different cell types can change during their consolidation, which makes it difficult not only to understand the phenomenon, but also to treat it. Advancing our knowledge of the molecular mechanisms underlying metastasis at each step holds fundamental importance for research to translate findings into novel, more personalized therapies to counteract metastasis.

## Figures and Tables

**Table 1:** Overview of studies on the influence of osteocytes on breast and prostate cancer cells.

Study	Effects on Breast Cancer	Effects on Prostate Cancer
Osteocyte-derived conditioned medium	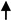 proliferation, migration, and invasion [41]	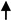 proliferation, migration, and invasion [41]
Tumor 3D spheroids grown in presence of osteocytes spheroids	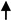 Tumor shrank [42] 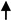 MET via down-regulation of Snail [43]	
3D microfluidic tissue	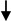 Cancer cells extravasation [45]	
	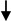 MMP9 expression and invasion of cancer cells [46] Interaction breast cancer cells/osteoclasts/endothelial cells [47]	
Mechanical loading of osteocytes (MLO)	Low level MLO: 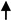 OPN, 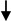 tumor migration; Medium level MLO: 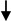 OPN, 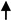 tumor migration [48]	
In vitro and in vivo experiments		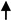 Growth and invasion via GDF15 [50]
Osteocytes under hydrostatic pressure	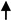 viability, invasion, and migration via RANTES and MMPs [51]	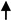 viability, invasion, and migration via RANTES and MMPs [51]

MET: mesenchymal-to-epithelial transition; MMP: metalloproteinases; MLO: mechanical loading of osteocytes; OPN: osteopontin; GDF15: growth-derived factor 15; RANTES: regulated upon activation, normal T cell expressed and secreted.
